# Incidental Discovery of Melanoma Metastasis in the Thenar Compartment: A Case Report

**DOI:** 10.7759/cureus.79405

**Published:** 2025-02-21

**Authors:** Mohammed Boubcheur, Samir Ben Salah, Abdeljaouad Najib

**Affiliations:** 1 Orthopaedics and Traumatology, Faculty of Medicine and Pharmacy of Oujda, Mohammed First University, Mohammed VI University Hospital, Oujda, MAR; 2 Faculty of Medicine and Pharmacy of Oujda, Mohammed First University, Mohammed VI University Hospital, Oujda, MAR

**Keywords:** hand tumors, incidental discovery, melanoma diagnosis, melanoma metastasis, orthopaedic hand surgery, secondary tumors

## Abstract

This report details the evaluation and management of a patient who presented with a tumor located in the thenar compartment of the left hand. The mass measured approximately 2 cm along its longest axis and exhibited no clinical signs of malignancy. However, imaging results and histopathological analysis of the biopsy indicated a malignant process, leading to the decision for tumor resection. The unique aspect of this case is the histopathological analysis of the surgical specimen, which revealed the tumor as a metastasis from a melanoma. This diagnosis was unexpected due to the location and clinical characteristics of the tumor. Biopsies of several dermatological lesions in the patient revealed a small lesion on the scalp, which was diagnosed as a localized melanoma.

## Introduction

Metastases to the hand are rare; they result most frequently from invasive tumors originating in various tissues, including the digestive, pulmonary, urological structures, or bone systems [1]. Therefore, in clinical practice, a mass in this region is generally not considered metastatic, particularly if the tumor shows no signs of malignancy and the patient has no history of cancer [2]. This often leads to misdiagnosis, underreporting, and delays in adequate management.

Melanomas of the hand are relatively rare, but a metastatic melanoma in the thenar aspect of the hand is exceptional. The terminal phalanges are the most frequent site of metastasis, followed by the metacarpals and proximal phalanges [3,4]. This presentation makes our case more unusual and confusing concerning the diagnosis and treatment.

## Case presentation

**Figure 1:**
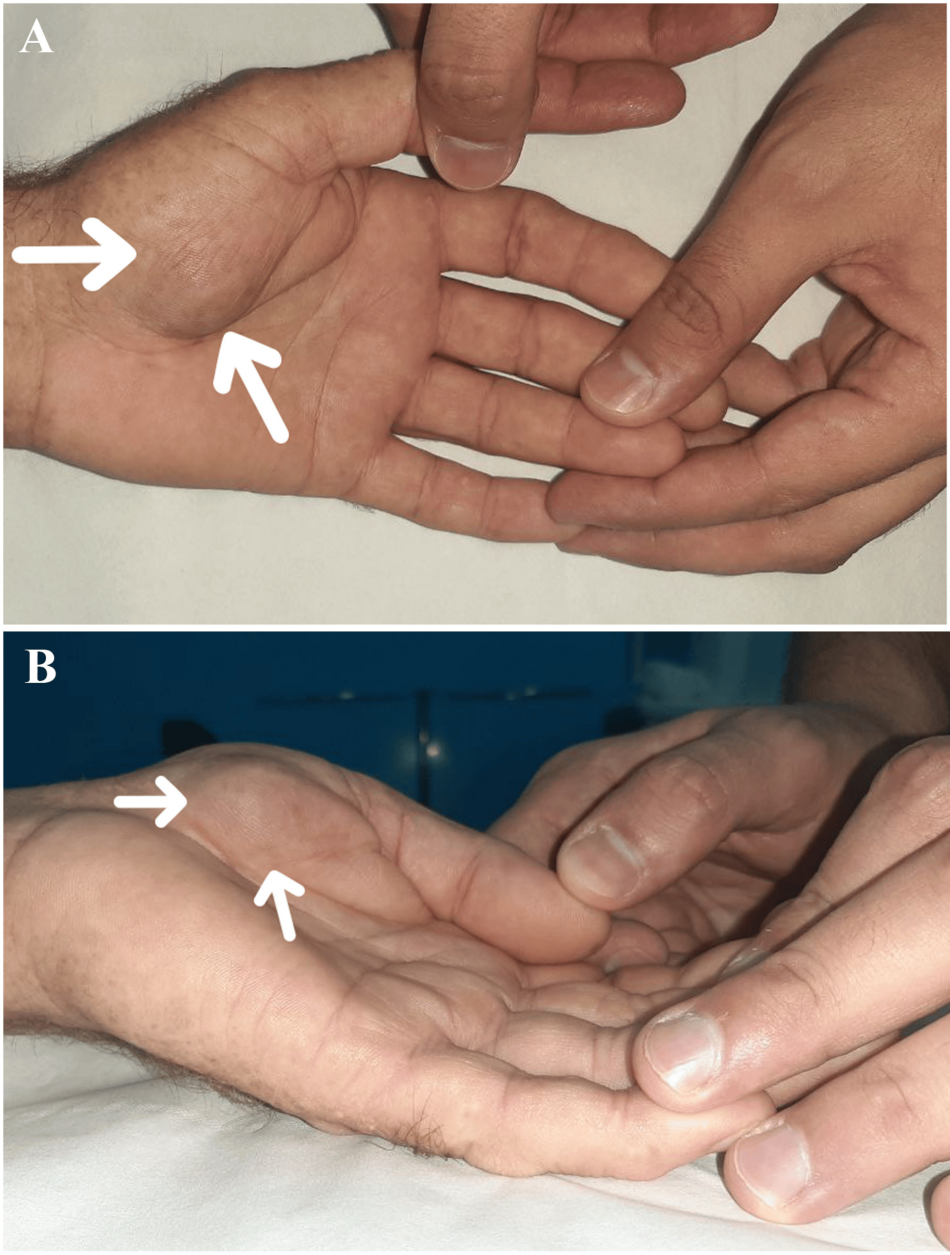
Clinical photograph of the tumor A: Frontal view; B: Lateral view

The patient was a 57-year-old man with no family history of skin cancer or other dermatological conditions. His occupation often required him to work outdoors, without using sunscreen. He exhibited a high total body naevus count and had a phenotype 1 characterized by red hair, freckles, and pale skin. He presented to our department with a painless swelling in the thenar region that had progressively enlarged over the past two years. The swelling was not accompanied by any systemic symptoms such as weight loss, anorexia, or general health decline. Clinical examination indicated the patient was stable and in good overall health. The mass, localized in the thenar region of the left hand, measured approximately 2 cm at its longest dimension, with no signs of local inflammation. The mass was mobile relative to superficial and deep planes, had a solid consistency, was painless on palpation, and did not impair hand function (Figure 1). Axial lymph nodes were not palpable.

**Figure 2:**
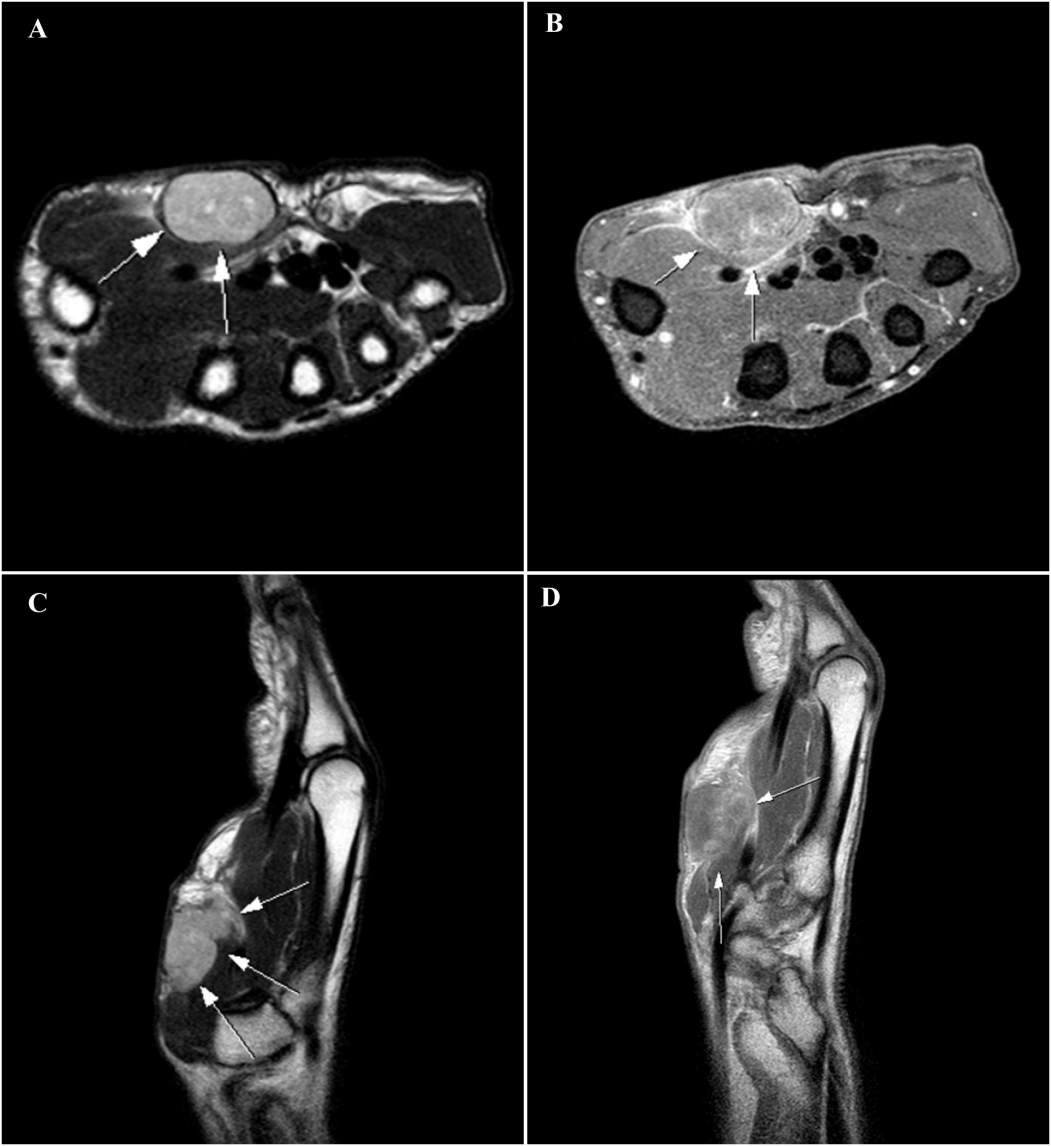
T2-weighted spin-echo sequences without fat saturation in the axial (A) and sagittal (C) planes demonstrate a nodular lesion in the subcutaneous layer of the palmar aspect of the hand. The intermediate signal intensity lesion is oval-shaped, with regular, well-defined margins. On T1-weighted spin-echo sequences with fat saturation in the axial and sagittal planes (B and D), the lesion shows mild contrast enhancement.

The patient underwent a soft tissue ultrasound of the hand, revealing a well-defined, heterogeneous mass within the thenar region. Subsequent MRI imaging suggested a tumor involving the superficial soft tissues of the thenar compartment. The MRI revealed small hemorrhagic areas, a pseudo-capsule rupture at the posterior wall, hypointensity on T1-weighted sequences, and infiltration of the thenar eminence muscle, with the median nerve and flexor tendons spared (Figure 2). The patient was admitted to the operating room for a biopsy under a medial-ulnar nerve block, with an incision made directly over the tumor. Biopsy results indicated a poorly differentiated carcinoma.

**Figure 3:**
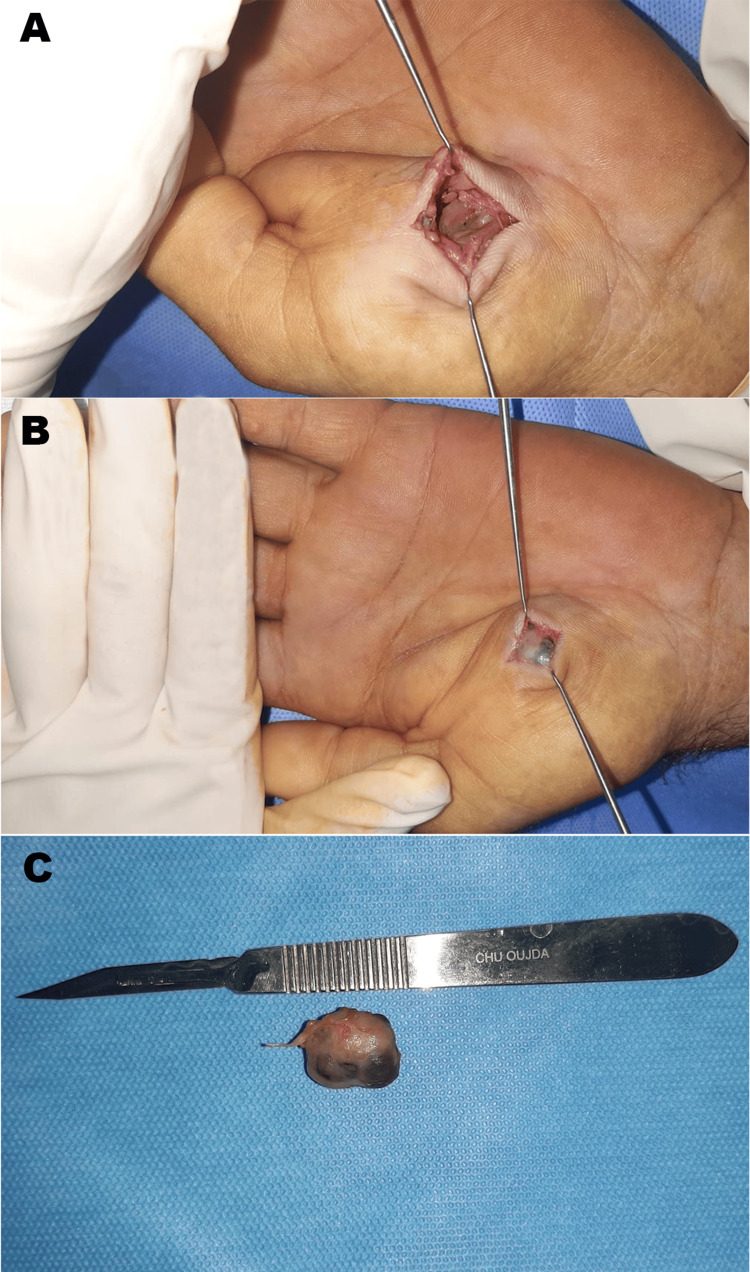
Intraoperative appearance of the tumor A: Tumor appearance after skin and subcutaneous incision; B: Tumor appearance following resection; C: Surgical specimen

An extended workup, including a cervico-thoraco-abdomino-pelvic CT scan and a brain MRI, revealed no abnormalities. A surgical decision was made to proceed with a carcinologic resection of the tumor under axillary block anesthesia. During surgery, the tumor appeared well-defined and encapsulated, enabling complete en-bloc resection (Figure 3).

**Figure 4:**
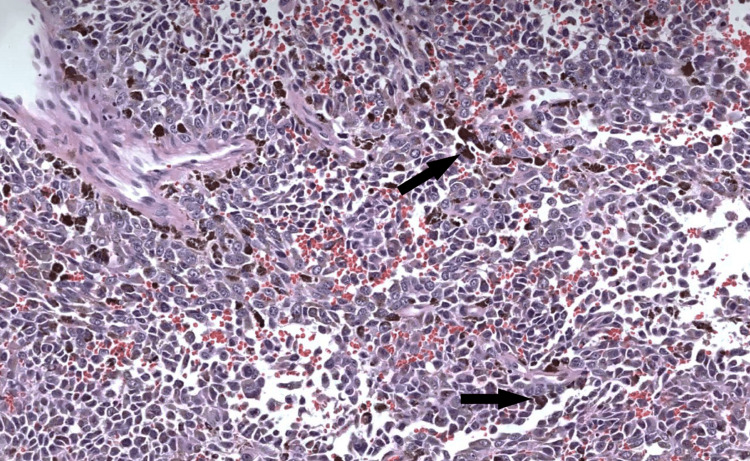
Photomicrograph of the lesion reveals a highly atypical proliferation composed of spindle to epithelioid cells with large nuclei and prominent nucleoli. The cytoplasm contains melanin pigments (arrows). Hematoxylin-Eosin (H&E): X200

The histopathological analysis of the tumor revealed a highly atypical proliferation composed of spindle to epithelioid cells with large nuclei and prominent nucleoli (Figure 4). The cytoplasm contains melanin pigments. The immunohistochemical study revealed that the tumoral cells were positive for HMB45, S100, and Melan-A, confirming the diagnosis of malignant melanoma. Invasion of vessels was not present in the resected lesion. All resection margins were safe.

Additionally, the patient presented with three small cutaneous nodules: one located at the nasal base, the second in the submental region, and the third in the left temporal region. Each nodule was excised and submitted for histopathological examination, which confirmed melanoma in the sample from the left temporal region.

He was referred to medical oncology for a specialized discussion of all treatment options, including chemotherapy. He received four cycles (every three weeks) of Dacarbazine as adjuvant therapy. After that, the patient declined any cancer-directed therapy and was unfortunately lost to follow-up at the time of this publication.

## Discussion

In this case, the tumor presented with clinically reassuring characteristics, with consultation sought primarily due to aesthetic concerns. However, imaging and histopathological biopsy analysis suggested a potentially malignant process. Metastasis was not initially suspected for several reasons: both the local and overall clinical presentation appeared benign, with the location of the tumor in the thenar region of the hand atypical for metastatic spread [5,6]. Metastatic lesions to the hand or wrist are rare and can mimic inflammatory or benign conditions such as gout or infections. This resemblance often leads to misdiagnosis, underreporting, and treatment delays. Tumors occurring in the hand, forearm, and arm often demonstrate unique growth patterns and metastatic potential compared to those in other anatomical sites [7,8].

A retrospective, descriptive meta-analysis was conducted on 2017 reviewed case files and literature from 1900 to 2017. Overall, 337 publications were analyzed, identifying 562 cases of hand metastases involving 480 patients. Sixty percent of the cases were male, with a mean age of 59 years. The most commonly reported primary cancers were lung (40%), gastrointestinal (19%), urological (13%), gynecological (11%), and head and neck (6%) cancers. The average survival time for patients was 7.2 months [9].

Of the metastatic case types, 59% were osseous, and 31% involved soft tissues. The thumb was the most commonly affected finger, followed by the middle finger, with a predominance of metastases in the distal phalanx, regardless of the primary cancer type. This case differs from those reported in the existing literature due to its unusual location in the thenar compartment and its primary origin as a melanoma, making it particularly noteworthy for discussion.

The prognosis of metastatic melanoma depends on multiple factors, including the stage of the disease, the presence of specific genetic mutations, and the patient's overall health status. The 5-year survival rate for patients with metastatic melanoma diagnosed between 2012 and 2018 was around 30%, highlighting the need for effective treatment options. Overall, the prognosis for patients with metastatic melanoma remains dismal, with a high mortality rate and limited adjuvant therapies. However, the development of targeted therapies and immunotherapies has significantly improved patient outcomes and survival rates [10].

## Conclusions

Metastatic melanoma of the hand is unfortunately a rare and challenging clinical entity. This case highlights the importance of maintaining a high level of awareness when evaluating unusual lesions in this region. Any hand tumor should be considered potentially malignant until proven otherwise, even in the absence of malignancy signs for prior tumor history in the patient to avoid misdiagnosis or delayed treatment. Melanoma, particularly in its metastatic form, is characterized by its aggressive nature and poor prognosis. Despite significant advances in therapeutic strategies, including immunotherapy and targeted therapies, it remains the leading cause of mortality from skin cancer and the death rate is still increasing worldwide. This underscores the critical need for early detection, accurate staging, and a multidisciplinary approach to management.

This report serves as a reminder of the unique challenges posed by metastatic melanoma of the hand and emphasizes the necessity for heightened awareness and prompt intervention to improve patient outcomes in these rare but severe cases.
